# Infiltrating macrophages in diabetic nephropathy promote podocytes apoptosis via TNF-α-ROS-p38MAPK pathway

**DOI:** 10.18632/oncotarget.18394

**Published:** 2017-06-07

**Authors:** Yinfeng Guo, Zhixia Song, Min Zhou, Ying Yang, Yu Zhao, Bicheng Liu, Xiaoliang Zhang

**Affiliations:** ^1^ Institute of Nephrology, Zhong Da Hospital, Southeast University School of Medicine, Nanjing, Jiangsu 210009, China

**Keywords:** macrophage, podocyte, apoptosis, diabetic nephropathy

## Abstract

Macrophage infiltration has been linked to the pathogenesis of diabetic nephropathy (DN). However, how infiltrating macrophages affect the progression of DN is unknown. Although infiltrating macrophages produce pro-inflammatory mediators and induce apoptosis in a variety of target cells, there are no studies in podocytes. Therefore, we tested the contribution of macrophages to podocytes apoptosis in DN. *in vivo* experiments showed that apoptosis in podocytes was increased in streptozocin (STZ)-induced diabetic rats compared with control rats and that this apoptosis was accompanied by increased macrophages infiltration in the kidney. Then, we established a co-culture system to study the interaction between macrophages and podocytes in the absence or presence of high glucose. Macrophages did not trigger podocytes apoptosis when they were co-cultured in the absence of high glucose in a transwell co-culture system. Additionally, although podocyte apoptosis was increased after high glucose stimulation, there was a further enhancement of podocyte apoptosis when podocytes were co-cultured with macrophages in the presence of high glucose compared with podocytes cultured alone in high glucose. Mechanistically, we found that macrophages were activated when they were exposed to high glucose, displaying pro-inflammatory M1 polarization. Furthermore, conditioned media (CM) from such high glucose-activated M1 macrophages (HG-CM) trigged podocytes apoptosis in a reactive oxygen species (ROS)-p38mitogen-activated protein kinases (p38MAPK) dependent manner, which was abolished by either a ROS inhibitor (Tempo) or a p38MAPK inhibitor (SB203580). Finally, we identified tumor necrosis factor (TNF-α) as a key mediator of high glucose-activated macrophages to induce podocytes apoptosis because an anti-TNF-α neutralizing antibody blunted the apoptotic response, excess ROS generation and p38MPAK activation in podocytes induced by HG-CM. Moreover, addition of recombinant TNF-α similarly resulted in podocytes apoptosis. In summary, the TNF-α that was released by high glucose-activated macrophages promoted podocytes apoptosis via ROS-p38MAPK pathway. Blockade of TNF-α secretion from high glucose activated macrophages and ROS-p38MAPK pathway might be effective therapeutic options to limit podocytes apoptosis and delay the progression of diabetic nephropathy.

## INTRODUCTION

Podocytes are considered critical cells in the development of diabetic nephropathy (DN) [1]. Reduction in podocytes number mediated by apoptosis has been observed in patients with both early and late DN [2–3] and in animal DN models [4]. The etiology of progressive disorder in podocytes is complex and multifactorial. Studies have suggested that macrophages played an important pathogenic role in this process [5].

Macrophage infiltration is an early response to renal damage, which precedes the decline of renal function and the onset of glomerular damage [6]. The number of macrophage infiltration is closely associated with the severity of proteinuria and histological damage in DN [7]. Glomerular macrophage depletion by diphtheria toxin has been shown to ameliorate podocytes injury in STZ-induced diabetic mouse model [8], thus further supporting a cell-cell interaction between macrophages and podocytes in diabetes mellitus. Recent studies indicated that macrophages are divided into M1 (classically activated macrophages) or M2 (alternatively activated macrophages) subtypes. M1 is characterized by excessive production of inducible nitric oxide synthase (iNOS), TNF-α and interleukin-1 (IL-1), whlie M2 exhibits increased mannose receptor (MR), arginase and IL-10 [9]. We and others have indicated that macrophages in the diabetic kidney were predominantly M1 phenotypes [10–11]. In addition, several studies have demonstrated that M1 macrophages rather than M2 macrophages destroy podocyte integrity and increase its permeability [8]. However, the effect of macrophages on podocytes apoptosis and the molecular mechanisms by which macrophages modulate the apoptosis response in podocytes remain poorly elucidated.

Reactive oxygen species (ROS) are crucial to the initiation of apoptosis in podocytes [12–13]. Previous studies have confirmed that ROS are over-expressed in podocytes in diabetic kidney diseases [14]. P38MAPK (p38mitogen-activated protein kinases), a pro-apoptotic signaling downstream of ROS, is subsequently activated, leading to cell apoptosis [15]. In addition, studies searching for the origin of increased ROS found that TNF-α, an effector molecule released by infiltrating macrophages, appears to be central for inducing ROS generation [16]. Therefore, we hypothesized that 1) infiltrating macrophages might promote podocytes apoptosis in DN and 2) mechanistically, TNF-α, a key mediator of macrophages, might account for the apoptotic response in podocytes via ROS-p38MAPK pathway.

## RESULTS

### Podocyte apoptosis in STZ-induced DN rats

**Figure 1:**
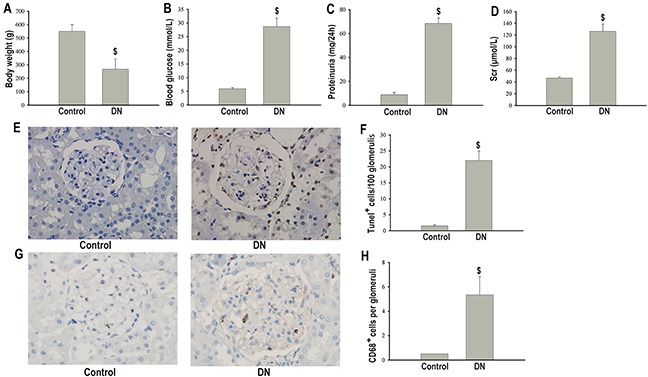
Podocytes apoptosis and macrophage infiltration in nondiabetic or STZ-induced diabetic rats at 18 weeks after STZ injection Body weight **(A)**. Blood glucose **(B)**. Proteinuria **(C)**. Scr **(D)**. TUNEL staining of apoptotic podocytes (×400) **(E)**. Semiquantitation of TUNEL positive cells per 100 glomerulis **(F)**. Immunohistochemical staining of CD68 positive macrophages in glomeruli (×400) **(G)**. Quantification of number of CD68 positive macrophages per glomeruli **(H)**. Data were presented as mean ± SD (n=8). ^$^P<0.05 vs. control group.

DN rats developed overt diabetes with higher blood glucose concentrations and lower body weights after the injection of STZ (Figure 1A, 1B). In addition, 24h-urinary proteinuria and serum creatinine (Scr) were markedly increased in diabetic rats than in non-diabetic rats (Figure 1C, 1D). Apoptosis in podocytes detected by TUNEL was significantly increased in the diabetic group compared with the non-diabetic group (Figure 1E, 1F).

### Macrophage infiltration in STZ-induced DN rats

Since inflammatory process has been proposed to contribute to the pathogenesis of DN, we assessed the degree of macrophage infiltration in diabetic glomeruli. Immumohistochemical staining showed that CD68 positive macrophages were markedly increased in the glomeruli of diabetic rats compared with non-diabetic controls (Figure 1G, 1H).

### Macrophages promoted podocytes apoptosis in the condition of high glucose

**Figure 2:**
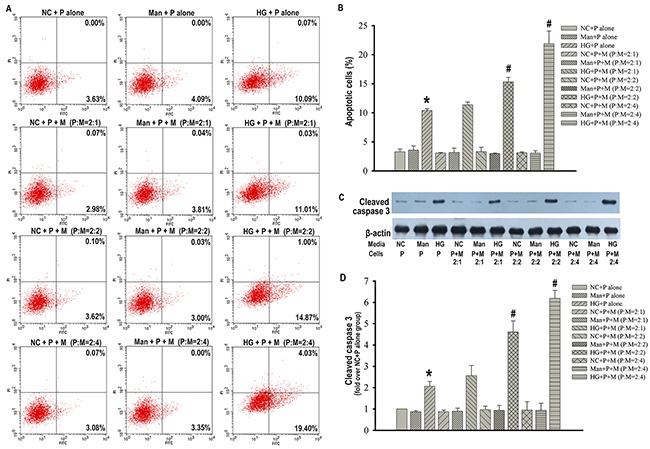
Macrophages promoted podocytes apoptosis in the condition of high glucose Flow cytometry analysis of podocytes apoptosis with Annexin V-FITC/PI staining **(A)**. Percentage of apoptotic podocytes **(B)**. Representative western blotting analysis of cleaved caspase 3 in podocytes **(C)**. β-actin was used as an internal control. Quantification of cleaved caspase 3 protein expression **(D)**. NC+P alone: podocytes treated with normal PRMI 1640 media. Man+P alone: Podocytes treated with 25 mM mannitol. HG+P alone: Podocytes treated with 25 mM high glucose. NC+P+M : Transwell co-culture of podocytes (P) and RAW 264.7 cells (M) in the absence of 25 mM high glucose at indicated ratios of P to M. Man+P+M: Transwell co-culture of P and M in 25 mM mannitol at indicated ratios of P to M. HG+P+M: Transwell co-culture of P and M in the presence of 25 mM high glucose at indicated ratios of P to M. Data were presented as mean ± SD from three independent experiments. *P<0.05 vs. NC+P alone group; ^#^P<0.05 vs. HG+P alone group.

The presence of macrophages in the injured kidney together with the increased apoptotic podocytes led us to examine the possibility that macrophages might promote the death of podocytes. Therefore, we co-cultured podocytes with macrophages in a transwell co-culture system in the absence or presence of high glucose, mimicking the diabetic microenvironment *in vivo*, and examined podocytes apoptosis. Interestingly, we found that macrophages did not trigger podocytes apoptosis when they were co-cultured in the absence of high glucose. Additionally, although both podocytes apoptosis and the apoptotic protein expression of cleaved caspase 3 were increased after high glucose stimulation, there was a further enhancement of podocytes apoptosis and cleaved caspase 3 expression when podocytes were co-cultured with macrophages in the presence of high glucose at podocytes (P): macrophages (M) ratios of 2:2 and 2:4 compared with podocytes cultured in high glucose alone (Figure 2). On the basis of the above results, we speculated that macrophages might be activated by high glucose and then promote podocytes apoptosis.

### Macrophages displayed pro-inflammatory M1 activation under high glucose condition

**Figure 3:**
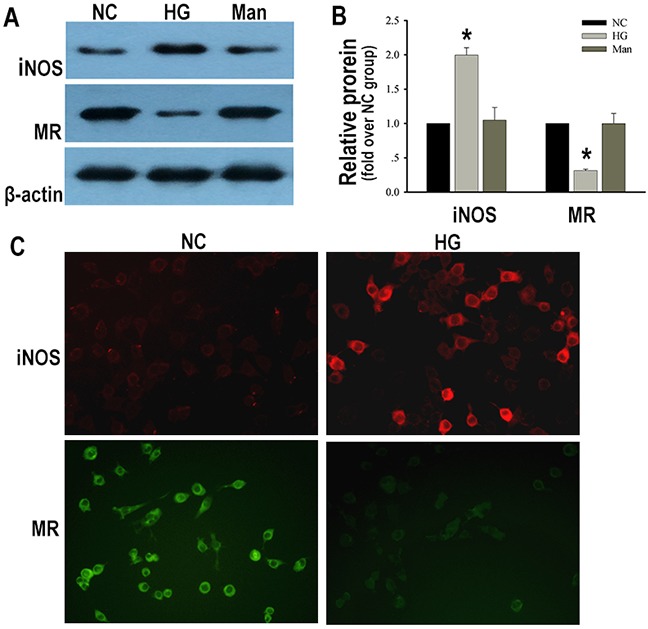
Macrophages displayed pro-inflammatory M1 activation under high glucose condition Representative western blotting analysis of iNOS and MR in macrophages **(A)**. Quantification of iNOS and MR protein expression in macrophages **(B)**. Immunofluorescent staining of iNOS and MR in macrophages (×200) **(C)**. NC: RAW 264.7 cells treated with normal glucose for 24 h. HG: RAW 264.7 cells treated with 25 mM high glucose for 24 h. Man: RAW 264.7 cells treated with 25 mM mannitol for 24 h. Data were presented as mean ± SD from three independent experiments. *P<0.05 vs. NC group.

Next, we detected macrophages phenotypes in the status of high glucose *in vitro*. The classical marker of M1 (iNOS) and specific marker of M2 macrophages (MR) [9] were measured. After stimulation with high glucose, macrophages increased their expression of iNOS. Additionally, MR was decreased when macrophages were exposed to high glucose compared with those exposed to normal glucose (Figure 3). These data indicated that macrophages displayed pro-inflammatory M1 activation in the condition of high glucose.

### Conditioned media from high glucose-activated macrophages (HG-CM) promoted podocytes apoptosis

**Figure 4:**
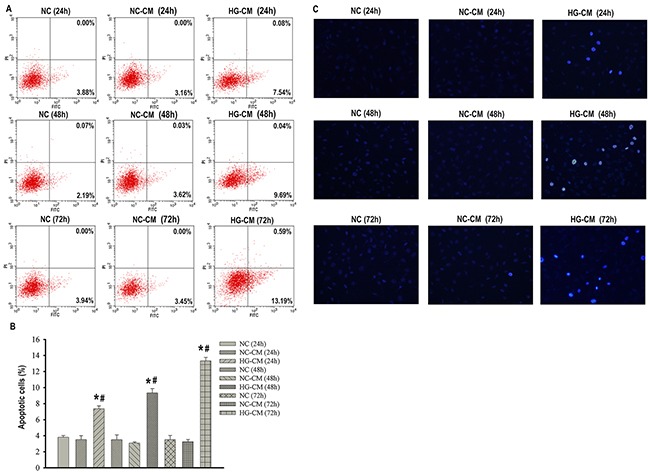
Conditioned media from high glucose-activated macrophages (HG-CM) promoted podocytes apoptosis Flow cytometry analysis of podocytes apoptosis with Annexin V-FITC/PI staining **(A)**. Percentage of apoptotic podocytes **(B)**. Hoechst-33342 staining of apoptotic podocytes (×200) **(C)**. NC: podocytes treated with norml RPMI 1640 media for indicated time. NC-CM: podocytes treated with NC-CM for indicated time. HG-CM: podocytes treated with HG-CM for indicated time. Data were presented as mean ± SD from three independent experiments. *P<0.05 vs. NC group; ^#^P<0.05 vs. NC-CM group at the same time point, respectively.

As high glucose activated pro-inflammatory M1 macrophages, it was of interest to determine whether such M1 macrophages were uniquely capable of promoting podocytes apoptosis. We collected CM from normal glucose-cultured macrophages (NC-CM) and from high glucose-activated macrophages (HG-CM) and then applied these media to podocytes. Meanwhile, podocytes treated with normal PRMI 1640 media were used as control. We found that there was no difference in podocytes apoptosis between control group and NC-CM group as showed by Annexin V-FITC/PI staining and Hoechst-33342 staining. Apoptosis in podocytes was significantly increased in a time-dependent manner when podocytes were exposed to HG-CM compared with either control or NC-CM (Figure 4), suggesting that such high glucose-activated macrophages directly promoted podocytes apoptosis by releasing a factor or factors.

### Apoptosis in podocytes triggered by high glucose-activated macrophages was ROS dependent

**Figure 5:**
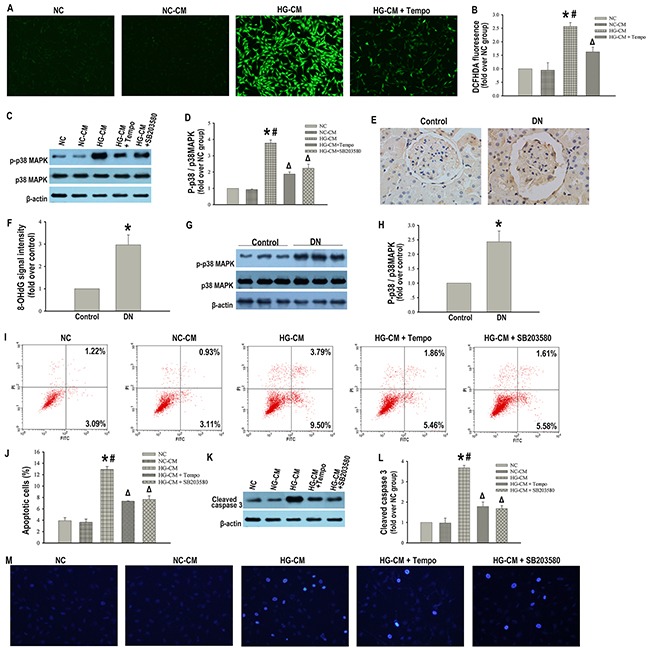
Apoptosis in podocytes triggered by high glucose-activated macrophages was ROS-p38MAPK dependent Representative DCFHDA staining of ROS in podocytes (×100) **(A)**. Quantitative analysis of ROS **(B)**. Representative western blotting analysis of p-p38MAPK and p38MAPK in podocytes **(C)**. β-actin was used as an internal control. Quantification of p-p38MAPK/p38MAPK in podocytes **(D)**. Immunohistochemical staining of 8-OHdG in glomerulus (× 400) of control or diabetic rats **(E)**. Semiquantitative analysis of 8-OHdG signal intensity **(F)**. Representative western blotting analysis of p-p38MAPK and p38MAPK in glomerular lysates of control or diabetic rats **(G)**. Quantification of p-p38MAPK/p38MAPK in glomerular lysates **(H)**. Data were presented as mean ± SD (n=8). *P <0.05 vs. control group. Flow cytometry analysis of podocytes apoptosis with Annexin V-FITC/PI staining **(I)**. Percentage of apoptotic podocytes **(J)**. Representative western blotting analysis of cleaved caspase 3 in podocytes **(K)**. Quantification of cleaved caspase 3 protein expression in podocytes **(L)**. Hoechst-33342 staining of apoptotic podocytes (×200) **(M)**. NC: podocytes treated with norml RPMI 1640 media. NC-CM: podocytes treated with NC-CM. HG-CM: podocytes treated with HG-CM. HG-CM + Tempo: podocytes treated with HG-CM and a ROS inhibitor (Tempo). HG-CM + SB203580: podocytes treated with HG-CM and a MAPK inhibitor (SB203580). Data were presented as mean ± SD from three independent experiments. *P<0.05 vs. NC group; ^#^P<0.05 vs. NC-CM group;^Δ^P<0.05 vs. HG-CM group.

Next, we investigated the mechanisms through which high glucose-activated macrophages induced podocytes apoptosis. Increased ROS has been reported to contribute to podocytes apoptosis in the progression of DN [17]. We then investigated whether ROS was involved in macrophages mediated podocytes apoptosis. We measured ROS level in podocytes cultured with HG-CM by DCFHDA analysis. There was no difference in ROS generation between the control group and the NC-CM group. However, ROS generation was significantly increased when podocytes were exposed to HG-CM (Figure 5A, 5B). Consistent with the results *in vitro*, 8-hydroxy-2-deoxyguanosine(8-OHdG) immunostaining also showed enhanced ROS in podocytes in STZ-induced diabetic rats (Figure 5E, 5F). Tempo, an inhibitor of ROS, markedly decreased HG-CM mediated ROS generation (Figure 5A, 5B). Moreover, Tempo reduced the proportion of apoptotic podocytes and cleaved caspase 3 expression elevated by HG-CM (Figure 5I–5M), thus validating a role for ROS in mediating the pro-apoptotic effect of high glucose-activated macrophages on podocytes.

### Inhibition of p38MAPK blunted podocytes apoptosis induced by high glucose-activated macrophages

We next tried to identify the molecular link between ROS activation and podocytes apoptosis induced by macrophages. It has been reported that excess ROS promoted cell apoptosis by p38MAPK pathway [15], We investigated whether the p38MAPK pathway was involved in high glucose-activated macrophages mediated podocytes apoptosis. The p38MAPK activation detected by the ratio of phosphorylated p38MAPK (p-p38MAPK) to total p38MAPK was markedly increased when podocytes were exposed to HG-CM compared with the ratios found in the control and NC-CM groups (Figure 5C, 5D). Consistent with this finding, p-p38MAPK/p38MAPK in kidney tissue was significantly increased in the diabetic rats compared with the control rats (Figure 5G, 5H). Furthermore, the phosphorylation effect of HG-CM on the p38MAPK in podocytes was blocked by either Tempo or a specific p38MAPK inhibitor (SB203580) (Figure 5C, 5D). In addition, SB203580 effectively decreased podocytes apoptosis and cleaved caspase 3 expression induced by HG-CM (Figure 5I–5M). These data suggested a direct link between ROS and p38MAPK, which accounted, at least in part, for the apoptotic response of podocytes triggered by high glucose-activated macrophages.

### Macrophage derived TNF-α is required to promote apoptosis in podocytes

**Figure 6:**
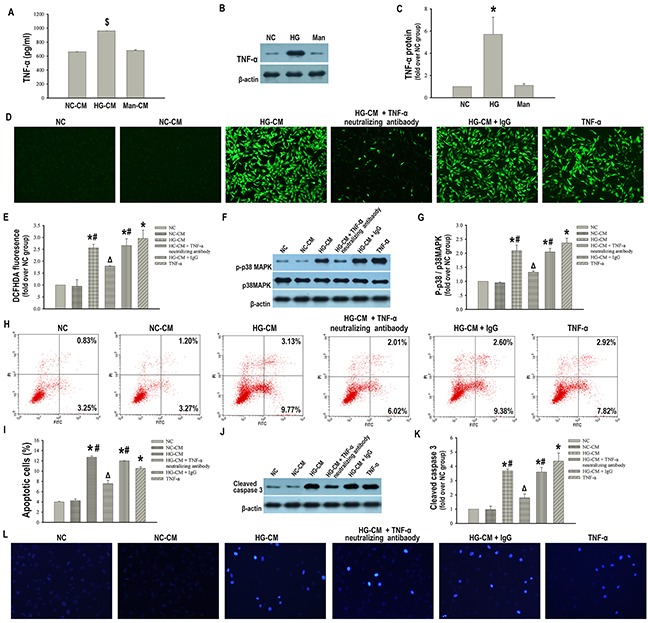
Macrophages derived TNF-α is required to promote apoptosis in podocytes ELISA assay of TNF-α level in the culture supernatant of macrophages **(A)**. Representative western blotting analysis of TNF-α in macrophages **(B)**. β-actin was used as an internal control. Quantification of TNF-α in macrophages **(C)**. NC-CM: CM from normal glucose-treated RAW 264.7 cells. HG-CM: CM from 25 mM high glucose-treated RAW 264.7 cells. Man-CM: CM from 25 mM mannitol-treated RAW 264.7 cells. NC: RAW 264.7 cells treated with normal glucose. HG: RAW 264.7 cells treated with 25 mM high glucose. Man: RAW 264.7 cells treated with 25 mM mannitol. Data were presented as mean ± SD from three independent experiments. ^$^P < 0.05 vs. NC-CM group; *P< 0.05 vs. NC group. Representative DCFHDA staining of ROS (×100) **(D)**. Quantitative analysis of ROS **(E)**. Representative western blotting analysis of p-p38MAPK and p38MAPK in podocytes **(F)**. Quantification of p-p38MAPK/p38MAPK in podocytes **(G)**. Flow cytometry analysis of podocytes apoptosis with Annexin V-FITC/PI staining **(H)**. Percentage of apoptotic podocytes **(I)**. Representative western blotting analysis of cleaved caspase 3 in podocytes **(J)**. Quantification of cleaved caspase 3 protein expression in podocytes **(K)**. Hoechst-33342 staining of apoptotic podocytes (×200) **(L)**. NC: podocytes treated with norml RPMI 1640 media. NC-CM: podocytes treated with NC-CM. HG-CM: podocytes treated with HG-CM. HG-CM + TNF-α neutralizing antibody: podocytes treated with HG-CM and anti-TNF-α neutralizing antibody. HG-CM + IgG: podocytes treated with HG-CM and IgG1. TNF-α: podocytes treated with TNF-α. Data were presented as mean ± SD from three independent experiments. *P< 0.05 vs. NC group; ^#^P < 0.05 vs. NC-CM group; ^Δ^P<0.05 vs. HG-CM group.

We have demonstrated that macrophages promoted podocytes apoptosis by releasing a factor or factors, which subsequently activated the pro-apoptotic ROS-p38MAPK pathway. Studies have demonstrated that TNF-α was a potent inducer of ROS production [18]. In particular, TNF-α derived from macrophages was recently described as a critical pathogenic factor in DN [19]. Therefore, we decided to focus on TNF-α. We speculated that macrophages derived TNF-α might be a key fuse for ROS-p38MAPK pathway and subsequent apoptosis in podocytes. First of all, we detected the level of TNF-α in the CM from macrophages, which was evidently increased in HG-CM compared with NC-CM (Figure 6A). Western blot analysis showed that protein expression of TNF-α was also increased when macrophages were exposed to high glucose compared with those cultured in normal glucose (Figure 6B, 6C). In addition, anti-TNF-α neutralizing antibody blunted the increased ROS production and p-p38MAPK/p38MAPK expression (Figure 6D–6G) and blocked the subsequent apoptotic rate by approximately 60% and cleaved caspase 3 expression in podocytes elicited by HG-CM (Figure 6H–6L), indicating TNF-α secreted from high glucose-activated macrophages was the essential factor in the above process. To unequivocally determine the function of TNF-α in podocytes apoptosis, we cultured podocytes with TNF-α alone and observed an obvious increase in apoptotic podocytes and cleaved caspase 3 expression (Figure 6H–6L). These data further argued for a pro-apoptotic function of this factor. Moreover, both ROS generation and p-p38MAPK/p38MAPK expression were increased when podocytes were exposed to TNF-α alone (Figure 6D–6G).

## DISCUSSION

The cell-cell interaction between infiltrating inflammatory cells and renal intrinsic cells has been the subject of extensive investigation in recent years in experimental inflammatory nephropathies, placing emphasis on the cross-talk between macrophages and renal cells such as mesangial cells, tubule cells and renal cell carcinoma cells with respect to cellular events such as proliferation, matrix synthesis, apoptosis and metastasis [20–22]. Our previous studies have demonstrated a profibrotic function of monocytes on proximal tubular epithelial cells (PTEC). Binding of monocytes via the intracellular adhesion molecule-1 (ICAM-1) expressed on the basolateral surface of PTEC stimulated the synthesis of TGF-β [23]. As podocytes are critical in maintaining glomerular function [24], several studies recently attempted to evaluate the role of macrophages on podocytes structure and function in DN despite the fact that they infiltrated in the glomeruli with diabetes in a low number, showing that macrophages had the potential to mediate podoctyes impairment [8]. However, the effect of macrophages on podocytes apoptosis remains poorly elucidated. Therefore, it is imperative to understand the cell-cell interaction between macrophages and podoctyes comprehensively and to identify effective therapeutic interventions and strategies to block the development of diabetic kidney disease.

In the current study, we firstly identified a novel correlation of macrophages with podocytes impairment in the progression of DN. Podocyte apoptosis was accompanied by an increased number of infiltrating macrophages in renal tissue in STZ induced diabetic rats. Next, we constructed a transwell co-culture system to characterize the intercellular communication between macrophages and podocytes. In such co-culture system, macrophages exerted a further pro-apoptotic effect on podocytes in the presence of high glucose but not in the absence of high glucose. Moreover, macrophages exhibited pro-inflammatory M1 phenotypes featured by increased cytokines such as iNOS and TNF-α when they were exposed to high glucose. Subsequently, we found that the CM from such high glucose-activated macrophages rather than inactivated macrophages promoted podocytes apoptosis via a panel of soluble molecules. Indeed, the requirement for macrophage activation to induce podocytes injury is consistent with other studies in which whether adoptively transferred macrophages could cause renal injury is dependent on their activation status [25]. Herein, we provided direct evidence of the pathogenic effect of high glucose-activated macrophages on podocytes apoptosis and loss in diabetic nephropathy.

How did high glucose-activated macrophages lead to podocytes apoptosis? Recent studies have painted an interesting picture on the emerging role of oxidative stress, whereby the excessive generation of ROS is associated with the structural and functional damage of various organs. Then, several intracellular transduction signal pathways are activated in response to oxidative stress, and regulate cell senescence, proliferation and apoptosis [15]. Data from animal and cell studies showed elevated levels of ROS accumulation in diabetic kidneys and renal parenchyma cells under multiple stimulus [15, 26]. Susztak et al. reported that the increased ROS generation was a potential mediator of podocytes apoptosis in DN. Mechanistically, elevated ROS induced podocytes apoptosis via the activation of pro-apoptotic p38MAPK pathway [4], which was also required for the induction of apoptosis by TGF-β in podocytes [27]. In the present study, we observed an increase of both ROS generation and its downstream signal molecule p-p38MAPK activation when podocytes were exposed to HG-CM but not NC-CM. In addition, podocyte apoptosis triggered by HG-CM was markedly reduced by an ROS inhibitor (Tempo) or a p38MAPK inhibitor (SB203580). These results indicated that high glucose-activated macrophages led podocytes to an apoptotic response via ROS-p38MAPK pathway. Moreover, we provided a new relationship between inflammation and oxidative stress that macrophages derived soluble molecules constituted another trigger for ROS generation in podocytes that are responsible for cells apoptosis and the progression of DN.

Activated macrophages secreting pro-inflammatory mediators is a vital event in the initiation of DN. TNF-α is increasingly considered as a potential mediator of macrophages and contributes to renal injury. Many animal and clinical studies have reported that TNF-α expression was increased in the urinary and diabetic kidney, which occureed prior to the rise in albuminuria and was independently correlated with clinical markers of glomerular and tubulointerstitial injury [28–29]. Inhibition of TNF-α with a neutralizing antibody reduced the features of DN in both type 1 and type 2 diabetes mellitus [19, 30]. However, TNF-α is produced not only by macrophages, but also by various renal resident cells such as mesangial cells and podocytes [31–32]. Therefore, the source of TNF-α that drives podocytes apoptosis in diabetic nephropathy remains unknown. Awad et al. demonstrated that specific deletion of TNF-α in macrophages significantly blocked both the basic and diabetes induced TNF-α levels and reduced albuminuria, plasma creatinine and histopathologic changes in diabetic animals [19]. Another study supporting a detrimental effect of macrophages derived TNF-α on cultured podocytes showed that this cytokine released by macrophages induced cytoskeleton reorganization [33]. In the present study, we found that the level of TNF-α was increased in the HG-CM. Anti-TNF-α neutralizing antibody abrogated podocyte apoptosis, excess ROS generation and p38MPAK activation elicited by HG-CM. Moreover, addition of TNF-α alone mimicked the pro-apoptotic effect of HG-CM on podocytes. Taken together, these data indicated that TNF-α released by high glucose-activated macrophages mediated podocytes apoptosis via TNF-α-ROS-p38MAPK pathway. However, an anti-TNF-α neutralizing antibody did not completely abolish ROS production, p38MPAK activation and subsequent apoptosis in podocytes, which suggests that other facts might also compensate for the apoptotic response in podocytes induced by high glucose-activated macrophages. Studies designed to identify these unknown mediators would provide more clues for understanding the exact role of macrophages in podocytes impairment in diabetic kidney disease.

The current study demonstrated that TNF-α secreted by infiltrating macrophages in diabetic kidney mediated a novel ROS-p38MPAK dependent apoptosis in podocytes. Blockade of TNF-α secretion from high glucose activated macrophages and ROS-p38MAPK pathway might be effective therapeutic strategies for limiting podocytes apoptosis and the progression of diabetic nephropathy.

## MATERIALS AND METHODS

### Animal experiments

The experimental protocol was approved by the Ethical Committee of Southeast University. Healthy male Sprague-Dawley rats weighing 200-220g were purchased from Shanghai Slac Laboratory Animal Co. Ltd (Shanghai, China). Rats were maintained under standard room temperature and regular 12h photoperiods, and were allowed free access to food and water. After one week of acclimatization, rats were randomly divided into two groups: (1) Control (n=8); (2) DN (n=8). DN was induced with a single intraperitoneal injection of streptozotocin (STZ, sigma) dissolved in 0.1 M citrate buffer (pH 4.5) at 58 mg/kg, while control rats received 0.1 M citrate buffer solution. Three days later, diabetic rats exhibited elevated blood glucose levels (≥16.7 mmol/L) by measuring tail blood glucose (BG) level. All animals were weighed and sacrificed at 18 weeks after diabetes was induced. Before being sacrificed, an individual rat was placed in metabolic cages to obtain 24 h urine. Blood samples were collected for biochemical studies, and kidneys were collected for histological examination and molecular assays.

### Macrophages culture

The mouse macrophage cells (RAW 264.7) were purchased from Shanghai Institutes for Biological Sciences (Shanghai, China). Cells were routinely maintained in normal RPMI 1640 media containing 11.1mM glucose supplemented with 10% fetal bovine serum (FBS), 100 U/ml penicillin and 100μg/ml streptomycin, and incubated at 37°C in 5% CO2. Conditioned media (CM) was prepared as described previously [33]. In brief, RAW 264.7 cells were seeded on six well-tissue culture plates, and incubated with or without 25mM high glucose for 24 h. The 25mM high glucose was achieved by adding 13.9 mM glucose to normal RPMI 1640 media (containing 11.1mM glucose). Then, the cells were washed three times to remove high glucose and debris, and cultured in normal RPMI 1640 media for a further 24 h. Then, these supernatants were collected, centrifuged at 2000rpm for 10 min and referred to as CM from normal glucose-cultured macrophages (NC-CM) or CM from 25mM high glucose-activated macrophages (HG-CM), respectively.

### Podocytes culture

The mouse conditionally immortalized podocyte cell line (kindly provided by Peter Mundel at the Mount Sinai School of Medicine, USA) between passage 14 and 20 was cultured on dishes coated with type I collagen (BD, USA) and in normal RPMI 1640 media supplemented with 10% FBS, 100 U/ml penicillin, and 100 U/ml streptomycin at 33°C in the presence of 10 U/ml mouse recombinant interferon-γ (Sigma, USA) and then differentiated for 10-14 days at 37°C in the absence of interferon-γ.

### Co-culture macrophages with podocytes

In a transwell co-culture system, RAW 264.7 cells (2×10^5^, 4×10^5^, 8×10^5^) seeded on a 0.4 μm Transwell insert (Millipore) were co-cultured with podocytes (4×10^5^) for 48 h in the absence or presence of 25 mM high glucose treatment. The ratios of podocytes (P) to macrophages (M) were 2:1, 2:2 and 2:4, respectively.

In the CM experiments, podocytes (4×10^5^) planted on six well plates were cultured overnight in normal RPMI 1640 media. Then, the cells were washed with PBS three times. After that, normal PRMI 1640 media (control), NC-CM or HG-CMwas added to podocytes for 24 - 72 h. In some experiments, 10μg/ml anti-TNF-α neutralizing antibody (RD, USA), 10μg/ml IgG1 Isotype control (RD, USA), 300μM ROS inhibitors (Tempo, sigma) or 10μM p38MAPK inhibitor (SB203580, RD, USA) was respectively added to cells with CM for 72 h. Besides, 10ng/ml recombinant mouse TNF-α (RD, USA) alone was applied to incubate podocytes for 72 h when necessary.

### Urine chemistry analysis

Scr was analyzed by an automatic biochemistry analyzer (Hitachi, Japan). 24 h-urinary proteinuria was measured using an ELISA Kit (Jian Cheng, China) according to the manufacturer's method.

### Immunohistochemical staining

Immunohistochemistry was performed on paraffin sections using a microwave based antigen retrieval technique. Sections were incubated with primary CD68 antibody (Santa Cruz, USA), 8-hydroxy-2-deoxyguanosine (8-OHdG, Santa, USA) antibody followed by appropriate secondary antibodies incubation. The immunostaining was visualized using DAB, and the slides were counterstained with hematoxylin. The mean number of CD68 positive macrophages was evaluated by counting in twenty randomly selected glomeruli. An immunohistochemistry semiquantitative analysis of 8-OHdG signal intensity was evaluated in twenty randomly selected areas using the Image-Pro Plus image analysis system.

### Measurement of apoptosis

A terminal deoxynucleotidyl transferase-mediated dUTP nick-end labeling (TUNEL) assay with an In Situ Cell Death Detection Kit (Roche, Germany) was performed to determine apoptosis within glomeruli following the manufacturer's protocol. Briefly, kidney tissues were fixed in 4% paraformaldehyde, incubated with TUNEL reaction mixture and then followed by anti-Fluorescein-POD conjugate. The degree of apoptosis was estimated using a scale based on the mean number of TUNEL-positive cells per 100 glomerular sections.

Annexin V-FITC/PI (BD, USA) staining was used to identify apoptotic cells *in vitro* according to the manufacturer's instructions. After being cultured with certain stimulation, cells were harvested, and centrifuged at 1200 rpm/min for 5 min, rinsed with PBS twice and resuspended in 400 μl 1× binding buffer containing 5μl PI and 5μl V-FITC, incubated for 15 min at the room temperature in the dark. The cell suspension was determined by flow cytometry to analyze the apoptotic rate. Cells in the upper-right quadrant and lower-right quadrant were classified as apoptotic.

Hoechst-33342 (Beyotime, China) staining was also used to evaluate podocyte apoptosis *in vitro*. After being fixed with 4% paraformaldehyde for 10 min at room temperature, these prepared cells were stained with Hoechst-33342 for 5 min at 37°C in the dark, and then washed with PBS three times. The Hoechst-stained nuclei characterized by nuclear condensation were visualized by fluorescence microscope.

### Immunofluorescent staining

Cells seeded on cover slips were fixed with 4% paraformaldehyde, permeabilized in 0.5% Triton-X100 for 30 min, and blocked with 1% BSA for 1h. After that, cells were washed and incubated with anti-mouse iNOS (Abcam, UK), MR (Abcam, UK) antibodies overnight at 4°C. Then, cells were incubated with a secondary antibody (Jackson, USA) for 2 h at room temperature and visualized using an IX70 fluorescence microscope (OLYMPUS, Japan).

### Reactive oxygen species (ROS) measurement

ROS was measured using 2′,-7′-dichlorodihydrofluorescein diacetate (DCFHDA, Sigma). Cells were incubated with DCFHDA at 37°C for 30 min and washed in PBS for 5 min. ROS generation in podocytes was visualized using a fluorescence microscope. The fluorescent intensity was measured by flow cytometry.

### ELISA

The TNF-α level in the CM was detected using ELISA kits (Neobioscience, China) according to the manufacturer's instructions.

### Western blot

The total proteins extracted from the renal cortex and cells were separated by sodium dodecyl sulfate -polyacrylamide gel electrophoresis (SDS-PAGE) and transferred to a nitrocellulose membrane. Nonspecific antibody binding was blocked by a preincubation of the membranes in 1×TBS containing 5% skim milk for 1h at room temperature. The membranes were incubated overnight with primary antibodies against iNOS (Santa, USA), MR (Abcam, UK), cleaved caspase-3 (Cell Signaling Technology, USA), p-p38MAPK, p38MAPK (Cell Signaling Technology, USA) at 4°C followed by incubation with horse reddish peroxidase (HRP) conjugated secondary antibodies for 1h. Finally, the membranes were visualized with an enhanced chemiluminescence advanced system (GE Healthcare, UK) and captured on X-ray film. Immunoreactive bands were quantified with densitometry using the Image J software (NIH, USA).

### Statistical analysis

All data were expressed as the mean ± standard deviation (SD) and analyzed with SPSS 16.0. Statistical differences among different groups were determined by one-way analysis of variance (ANOVA). A difference was considered significant if the P value was less than 0.05.
